# A Rapid Flow Cytometric Antimicrobial Susceptibility Assay (FASTvet) for Veterinary Use – Preliminary Data

**DOI:** 10.3389/fmicb.2020.01944

**Published:** 2020-08-07

**Authors:** Ferdinando F. Andrade, Rosário Gomes, Inês Martins-Oliveira, Ana Dias, Acácio G. Rodrigues, Cidália Pina-Vaz

**Affiliations:** ^1^Department of Microbiology, Faculty of Medicine, CINTESIS, University of Porto, Porto, Portugal; ^2^Farmanimal Veterinary Centre, Caldas da Rainha, Portugal; ^3^FASTinov, Porto, Portugal; ^4^CINTESIS – Center for Health Technology and Services Research, Faculty of Medicine, University of Porto, Porto, Portugal

**Keywords:** antimicrobial resistance, antimicrobial susceptibility test, veterinary, flow cytometry, one health concept

## Abstract

A rapid flow cytometric antimicrobial susceptibility test for bacteria isolated from companion animals – the FAST*vet* assay, developed by FASTinov^®^, was evaluated. Bacterial strains isolated from different biological samples of companion animals with infectious diseases in progress were obtained from several veterinary clinical laboratories across the country. A total of 115 strains, comprising 65 Gram-negative and 50 Gram positive isolates, were incubated with 13 antimicrobial drugs (ampicillin, amoxicillin-clavulanic acid, piperacillin-tazobactam, cefpodoxime, imipenem, enrofloxacin, gentamicin, amikacin for Gram-negative; penicillin, cefoxitin, enrofloxacin, vancomycin and ampicillin for Gram-positive) at breakpoint concentrations following CLSI protocol (CLSI Vet 01, 2018) for 1 h and analyzed by flow cytometry. The overall categorical agreement was 95.6% in case of Gram-negative and of 96.7% in Gram-positive isolates when compared to microdilution. FAST*vet* kits contribute to reduce the turnaround time (2 vs. 24 h) with early determination of the antimicrobial susceptibility profile. The correct and rapid choice of the target antibiotic therapy, will have a positive impact on animal care, contributing for preventing antimicrobial resistance. In conclusion, FASTinov^®^ vet kits showed an excellent performance, both for Gram-negative and Gram-positive isolates encouraging us to enlarge the sample size and planning multicentric studies.

## Introduction

The availability of effective antimicrobials has enabled considerable advancements in modern medical and veterinary practice that would have been unachievable without them. Antimicrobial drugs are a precious resource daily used all over the world to treat infections both in humans and animals. Increased antimicrobial resistance due to unnecessary use of antibiotics is a major obstacle to keep these drugs useful for as long as possible. Microbial multidrug resistance is emerging worldwide at an alarming rate and is now recognized as a major health threat (Infectious Diseases Society of America, 2011).

There is a growing concern regarding both public health and animal welfare about the consequences of antimicrobial resistance (AMR) in bacteria from animal sources. The appropriate use of antimicrobial drugs (AMDs) in veterinary medicine is one of the key areas of European Union (EU) policy objectives to combat AMR. Various initiatives have been started by many national, European and international bodies to promote a wise use of AMDs both in human and veterinary medicine. The importance of accurate and rapid laboratorial diagnostics regarding Antimicrobial Susceptibility Testing (AST) as the basis for a rational prescription of antimicrobials to treat an infection has been advocated by numerous international and national guidelines and regulatory agencies. AST is considered as one of the most important factors governing the adequate selection of antimicrobials, both for human and veterinary use. The primary objective is the target selection of the most appropriate antibiotic, the second involves a better control of public health hazards and the third objective is the provision of valid epidemiological AST surveillance data.

The early start of effective antibiotic therapy during the course of an infectious disease reduces the probability of pathogens developing AMR (Deresinski, 2007). Particularly in sepsis cases and bloodstream infections, early and targeted antimicrobial therapy is also crucial to decrease mortality rates (Kumar et al., 2006). Empirical antimicrobial therapy is often started as soon as possible, especially on sepsis management without waiting for AST results (Cohen, 2009; Dellinger et al., 2013). In most of such cases, therapy involves the selection of broad-spectrum antibiotics or a combination of antibiotics which promotes the emergence of resistance (Micek et al., 2010). Early assessment of AST is therefore essential for statement of targeted antimicrobial therapy in order to avoid the escalating rates of resistance and to decrease mortality. At present, in routine clinical microbiology laboratories, ASTs is usually assessed by automated systems such as Vitek2 (BioMérieux), MicroScan Walkaway (Beckman Coulter) or Phoenix (Becton Dickinson) (Mittman et al., 2009; Duggal et al., 2012; Gherardi et al., 2012); or semi-automated method like Sensititre (Thermofisher) (Rene, 2010); in alternative, manual tests such as disc diffusion, broth dilution or Etest can be used (Jenkins and Schuetz, 2012; Van Belkum et al., 2013). While a diversity of tests can be performed, all of them are based upon the study of the growth ability of microorganisms which takes at least 24 h to test results.

Recently, several technologies have been pointed out as more rapid alternatives for ASTs such as whole-genome sequencing, mass spectrometry, fluorescence-activated cell sorting and microarrays particularly useful regarding the detection of known mechanisms of resistance (Van Belkum et al., 2013). However, such approaches use very expensive instruments and procedures, resulting in high analysis costs. In addition, they can only detect previously known mechanisms of resistance. In clinical practice, human or veterinary, faster AST methods are required in order to get an effective treatment, applying the most suitable antibiotic as soon as possible, ideally within the same day. We validated a novel methodology of AST involving the use of flow cytometry (FC), using microbial isolates obtained from companion animals with infectious diseases in progress. This is a disruptive approach for rapid AST. FC allows single cell analysis and is a very fast and accurate method (Andrade et al., 2018). This study was conducted in collaboration with FASTinov^®^, a spin-off company of Porto University, Portugal from June 2018 to June 2019, the company responsible for FASTvet kit manufacturing.

## Materials and Methods

### Bacterial Strains

A total of 65 Gram-negative bacilli (40 *E. coli*, 10 *Klebsiella pneumoniae*, 5 *Proteus mirabilis*, 8 *Salmonella* spp., 2 *Pasteurella multocida*), 30 *Staphylococcus* spp. (10 *S. aureus*, 10 *S. epidermidis* and 10 *S. pseudintermedius*) and 20 *Enterococcus* spp. (14 *E. faecalis* and 6 *E. faecium*) isolated from various species of companion animals with infectious diseases in progress and from different biological samples (skin soft tissue, cyst content, infected wounds) were made available by several microbiological veterinary laboratories across the country. In addition, type strains belonging to American Type Culture Collection (ATCC): *Escherichia coli* 35218, *E. coli* 25922; *P. aeruginosa* 27853; *Staphylococcus aureus* 29213, *S. aureus* 43300, *Enterococcus faecalis* 29212, *E. faecalis* 51299 and *E. faecium* 700221 were also included in this study.

### FASTvet Panels

Two AST panels were previously developed and tested by FASTinov^®^ in a microplate format: the FAST*vet* gramneg (for Gram-negative bacilli) and the FAST*vet* grampos (for *Staphylococcus* and *Enterococcus*). Antibiotics (see Table 1), at drug breakpoint concentrations adequate to the origin of the bacterial strain (see Table 2), were selected according the CLSI vet protocol from 2018 (CLSI 5th Edition, 2018). A fluorescent probe is also present in each well. Distinct probes, such as nucleic acid staining or membrane depolarization status, previously optimized, were selected in order to reveal distinct cell lesions produced by antimicrobials.

**TABLE 1:** Susceptibility phenotypes evaluated using reference methods according CLSI, comparison with flow cytometry assay.

				BMD			FASTinov versus BMD			
	Antimicrobial	n	MIC intervale μg/ml	S	I	R	mE	ME	VME	CA
Gram negative bacilli	Ampicillin	65	1–≥64	15	–	50	0	0	0	100%
	Amoxicillin-clavulanic acid	65	0.06/4–≥32/4	16	1	48	2	3	0	92.3%
	Piperacillin-tazobactam	65	≤0.125/4–≥64/4	56	1	8	4	0	0	93.9%
	Cefpodoxime	65	0.06–≥64	52	1	12	0	0	0	100%
	Imipenem	65	0.25–≥64	60	3	2	3	1	0	93.9%
	Enrofloxacin	65	≤0.06–≥32	46	2	17	2	1	0	95.4%
	Gentamicin	65	≤0.125–≥32	56	2	7	3	1	0	93.9%
	Amikacin	65	0.06–64	58	2	5	3	0	0	95.4%
	Overall	520		359	12	149	17	6	0	95.6%
*Staphylococcus* spp.	Penicillin	30	≤0.06–36	6	–	24	0	1	0	96.7%
	Cefoxitin	30	0.25–8	23	–	7	1	1	0	93.3%
	Enrofloxacin	30	≤0.06–≥32	28	–	2	1	0	0	96.7%
	Vancomycin	30	0.06–1	30		–	2	0	0	93.3%
*Enterococcus* spp.	Ampicillin	20	1–8	16	2	2	0	0	0	100%
	Vancomycin	20	0.5–8	16	2	2	0	0	0	100%
	Overall	180		135	6	39	4	2	0	96.7%

*S-susceptible; I-intermediate and Resistant. Categorical agreement (CA) and number of errors: mE-minor errors; ME- major errors and VME-very major errors.*

**TABLE 2:** Antimicrobial concentrations included on FAST*vet* (breakpoint values according CLSI).

Antimicrobial		FAST*vet* panel concentrations (μg/ml)				
	Phenotype	S	I	R	S (urine)	R (urine)
Gram negative bacilli	Ampicillin	≤0.25	0.5	>0.5	≤8	>8
	Amoxicillin-clavulanic acid	≤0.25/0.12	0.5/0.25	>0.5/0.25	≤8/4	>8/4
	Piperacillin-tazobactam*	≤16/4	64/4	>64/4		
	Cefpodoxime	≤2	4	>4		
	Imipenem* *Enterobacterales*	≤1	2	>2		
	Imipenem* *Pseudomonas* spp.	≤2	4	>4		
	Enrofloxacin *Enterobacterales*	≤0.12	0.25	>0.25		
	Enrofloxacin *Pseudomonas* spp.	≤0.5	2	>2		
	Gentamicin	≤2	4	>4		
	Amikacin	≤4	8	>8		
*Staphylococcus* spp.	Penicillin*	≤0.12		>0.12		
	Cefoxitin*	≤4		>4		
	Enrofloxacin	≤0.5	2	>2		
	Vancomycin* *S. aureus*	≤2	8	>8		
*Enterococcus* spp.	Vancomycin* *S.* coagulase neg	≤4	16	>16		
	Ampicillin*	≤8		>8		
	Vancomycin*	≤4	16	>16		

*Human breakpoints. According to flow cytometry analysis when the cells show lesions with the lowest concentration tested, the strain is considered S (susceptible); if no lesion is shown with the lower concentration and but only with the second concentration, the strain is considered I (intermediate); if even with the highest concentration no effect is seen, the strain is considered R (resistant). The category of I is not always present.

### Inoculation and Incubation of the AST Panels

Pure colonies isolated in solid media, were inoculated in Brain-Heart broth cation adjusted (Sigma) and incubated at 37°C shaking until turbidity (aprox. 1 h), in order to obtain an exponential growth phase culture (Faria-Ramos et al., 2013); after a 2 centrifugation step, a microbial suspension was obtained and adjusted to the optimized concentration of cells and the FAST*vet* gramneg or FAST*vet* grampos panels inoculated accordingly to the microorganism. FAST*vet* panels were then incubated during 1 h, protected from light, at 37°C, shaking 540 rpm/min. Repeatability and reproducibility were determined by testing 10 strains in triplicate, from independent inoculums, which MIC values are on-scale, according ISO 20776-2 (ISO 20776-2, 2016).

### Flow Cytometric Analysis

The FAST*vet* panels were analyzed in a CytoFLEX flow cytometer (Beckman Coulter) using previously optimized templates. The number of cells on the “gate”- zone of analysis; the complexity of the cells (measured by the side scatter); size of the cells (measured by the forward scatter) and the intensity of fluorescence of the cells are the 4 main parameters analyzed. Treated cells are always compared with non-treated cells for each tested microbial strain. A dedicated software, the BioFAST^®^, developed for AST for human health, was adapted to this veterinary assay. Antibiotic susceptibility was determined by the use of an algorithm in the BioFAST software (FASTinov^®^, Porto, Portugal) that determines the ratio between the mean fluorescence intensity (MFI) of treated bacteria compared with control cells (non-treated bacteria); this ratio was calculated through ROC curves and defined as the staining index (SI). For each antibiotic and bacterial combination, cut-off values were incorporated into the BioFAST software for antimicrobial susceptibility testing.

The available prototype provides a stand-alone application with limited licensing support, interacting with flow cytometers using a commonly supported file format, which most cytometers export.

### Reference AST Method

All tested strains were analyzed through reference methods, either microdilution or disk diffusion tests, according to CLSI AST vet document (CLSI 5th Edition, 2018) and classified as susceptible (S), intermediate (I) or resistant (R). Recommended QC were included.

### Data Analysis and Comparison With Reference AST Methods

Taking into consideration the classification of the AST phenotypes determined by reference methods, the cut-off values for the cytometric assay were calculated using ROC curves and included in the BioFAST vet software. The phenotype determined using the FAST*vet* assay was therefore recorded and compared with the obtained with reference methods. In case of discrepancies, the susceptibility test was repeated by both methods. Categorical agreement was calculated and errors were classified as very major errors (VMEs; a false susceptible), major errors (ME; a false resistant) and minor errors (mEs; any false result involving an intermediate result) according to International Organization for Standardization [ISO] (20776), 2006 part 2.

### Statistical Analysis

The percentage agreement (PA) was calculated in order to evaluate repeatability and reproducibility.

## Results

A total of 115 microbial isolates were included in the present study, including 65 Gram-negative isolates and 50 Gram-positive strains. The overall categorical agreement (CA) between the new cytometric AST assay and the reference method was 95.6% (range between 92.3–100%) for FAST*vet* gramneg and 96.7% (range between 93.3–100%) on FASTgrampos. CA and errors distribution is detailed on Table 1, no VM errors were found.

**FIGURE 1:**
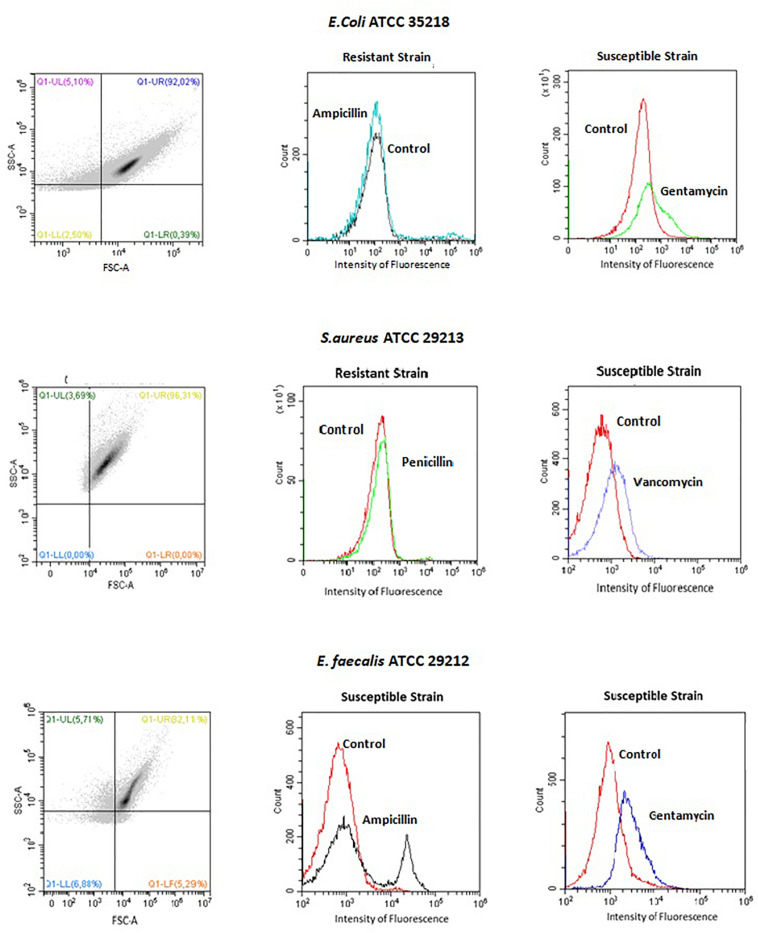
Dot plot and histograms of a typical flow cytometry analysis of one Gram negative bacilli (*E. coli* ATCC 35218) and two Gram positive cocci (*S. aureus* ATCC 29213 and *E. faecalis* ATCC 29212). Note an increase on the intensity of fluorescence on the susceptible strains.

Typical dot plots and histogram are shown in Figure 1, as examples. An increase of the intensity of fluorescence (moving to the right) of the drug-treated microbial population comparing with non-treated cells (control), means that the cells depolarized, corresponding to a susceptible phenotype. In such strains, a decrease of the number of cells (cell counts) is common. Resistant phenotypes, reveal similar pattern to the control (no changes on fluorescence or reduction on cell number was seen). An intermediate phenotype is detected whenever an increase of the intensity of fluorescence is observed only following incubation with the highest concentration of the drug (the lowest concentrations produce no effect). Regarding reproducibility, 96.6% was obtained (PA = 0.966) and the repeatability was 100% (PA = 1).

The time required to obtain a final AST, using FAST*vet* panel was 2 h, in contrast with the minimum of 24 h required by the current standard AST methods, thus resulting in a gain in time to result of 22 h in average.

## Discussion

The purpose of this study was to evaluate the performance of FAST*vet* (flow cytometry antimicrobial susceptibility test for veterinary) and to determine whether this procedure could be used routinely to reduce AST turnaround time in veterinary medicine. This will allow the early choice of targeted antibiotic therapy, by the veterinary surgeon whenever managing an animal with an infectious disease in progress. This methodology was first applied to human health and, attending to the many similitudes of microorganisms/drugs required, we proposed it could be transferred, with similar advantages, to veterinary field. The One Health Concept stresses in particular, the problem of antimicrobial resistance that is global and affects both human and animals (American Veterinary Medical Association, 2008; Andrade et al., 2018). Among the various alternatives and contingencies considered to prevent the emergence of resistance to antimicrobials, a fast and accurate AST is urgently needed. This study demonstrates the potential utility of FAST*vet* as a useful AST tool to assist the veterinary surgeon in clinical useful time in the selection of targeted antimicrobials (Andrade et al., 2018) and supports further investigation and validation on this test methodology. A quick and accurate clinical treatment would prevent the dissemination of multidrug-resistant bacteria.

Pure colonies were used in this study and, around 1 h subculture in a broth was needed especially for β-lactamic drugs since they only act when cells reach the exponential growth phase. However, such a FC assay could be started not only with colonies but also directly from positive blood cultures (Costa-de-Oliveira et al., 2017) or in urine (data not shown) as there are enough cells on exponential growth phase. This will avoid the sub-culture step fast-tracking the answer to a maximum of 2 h instead of 2 days.

A limitation of this technology is that, like all other phenotypic AST tests, it cannot be performed directly from polymicrobial products or mixed cultures. In such instances, molecular tests could detect a resistance gene, e.g., mecA in a nasal swab meaning the presence of a MRSA. Nevertheless, only some genes could be searched for detection of resistance and its absence does not guarantee susceptibility. Another limitation is the fact that this test does not provide minimum inhibitory concentrations (MIC values), as only breakpoint concentrations were included. Flow cytometry assay could provide MIC values but we privileged the speed (more concentrations will increase the time of analysis) and few clinical situations require that information. By contrary, quantitative values could be very relevant for epidemiological purpose but, in that case, it would be better to calculate epidemiological cut-off values (ECV) than MIC.

With the present study, we demonstrate that AST results for veterinary antimicrobials could be obtained within the same working day, about 24 h earlier than results obtained with the usual standard diagnostic procedure. The performance of the FAST*vet* kit agrees with the ISO recommendations for AST. Those results are promising and lead us to increase the sample size. An external validation, in dedicated veterinary labs, is being planned. This new and disruptive technology could change the clinical diagnostic paradigm in relation to AST in veterinary medicine.

## Data Availability Statement

The raw data supporting the conclusions of this article will be made available by the authors, without undue reservation.

## Author Contributions

FA wrote the manuscript, collected the biological samples, and designed the study. RG and IM-O executed the experimental work. AR wrote the manuscript and designed the study. CP-V wrote the manuscript and designed the study and coordinated the research. All authors contributed to the article and approved the submitted version.

## Conflict of Interest

RG, IM-O, AD, and CP-V were employed by FASTinov. The remaining authors declare that the research was conducted in the absence of any commercial or financial relationships that could be construed as a potential conflict of interest.
